# Dissecting Genomic Aberrations in Myeloproliferative Neoplasms by Multiplex-PCR and Next Generation Sequencing

**DOI:** 10.1371/journal.pone.0123476

**Published:** 2015-04-20

**Authors:** Martin M. J. Kirschner, Mirle Schemionek, Claudia Schubert, Nicolas Chatain, Stephanie Sontag, Susanne Isfort, Nadina Ortiz-Brüchle, Karla Schmitt, Luisa Krüger, Klaus Zerres, Martin Zenke, Tim H. Brümmendorf, Steffen Koschmieder

**Affiliations:** 1 Department of Hematology, Oncology, Hemostaseology, and Stem Cell Transplantation, Medical Faculty, RWTH Aachen University, Aachen, Germany; 2 Institute for Biomedical Engineering—Cell Biology, RWTH Aachen University, Aachen, Germany; 3 Institute for Human Genetics, Medical Faculty, RWTH Aachen University, Aachen, Germany; Queen's University Belfast, UNITED KINGDOM

## Abstract

In order to assess the feasibility of amplicon-based parallel next generation sequencing (NGS) for the diagnosis of myeloproliferative neoplasms (MPN), we investigated multiplex-PCR of 212 amplicons covering genomic mutational hotspots in 48 cancer-related genes. Samples from 64 patients with MPN and five controls as well as seven (myeloid) cell lines were analyzed. Healthy donor and reactive erythrocytosis samples showed several frequent single-nucleotide polymorphisms (SNPs) but no known pathogenic mutation. Sequencing of the cell lines confirmed the presence of the known mutations. In the patient samples, JAK2 V617F was present in all PV, 4 of 10 ET, and 16 of 19 MF patients. The JAK2 V617F allele burden was different in the three groups (ET, 33+/-22%; PV 48+/-28% and MF 68+/- 29%). Further analysis detected both previously described and undescribed mutations (i.e., G12V NRAS, IDH1 R132H, E255G ABL, and V125G IDH1 mutations). One patient with lymphoid BC/Ph+ ALL who harbored a T315I ABL mutation and was treated with ponatinib was found to have developed a newly acquired V216M TP53 mutation (12% of transcripts) when becoming resistant to ponatinib. Ponatinib led to a decrease of ABL T315I positive transcripts from 47% before ponatinib treatment to 16% at the time of ponatinib resistance in this patient, suggesting that both TP53 and ABL mutations were present in the same clone and that the newly acquired TP53 mutation might have caused ponatinib resistance in this patient. In conclusion, amplicon-sequencing-based NGS allows simultaneous analysis of multiple MPN associated genes for diagnosis and during treatment and measurement of the mutant allele burden.

## Introduction

Myeloproliferative neoplasms (MPN) are a heterogeneous group of hematopoietic disorders which arise from genetically altered myeloid stem or progenitor cells. MPN are characterized by an overproduction of mature myeloid cells, which is due to deregulated cell-autonomous proliferation and which may result in splenomegaly and constitutive symptoms. In addition to the affected hematopoietic lineage (granulocytes, eosinophils, mast cells etc.), molecular markers are used to classify MPN subtypes. Since the first description of the Bcr-Abl oncogene, molecular genetic tools have become instrumental in diagnosis and treatment monitoring of MPN. In addition to the development of highly sensitive quantitative polymerase chain reaction (PCR) for RNA expression analysis, fundamental improvements have been made in the field of automated sequencing. Sanger sequencing, having dominated the field for decades, is now complemented by “next generation sequencing” (NGS). These methods allow for a fast, sensitive, and cost-efficient high-throughput screening of genomic aberrations and their application has already fundamentally improved our understanding of how genetic alterations affect health and disease. For classical Philadelphia chromosome-negative (Ph^-^) MPN, which comprise Essential Thrombocythemia (ET), Polycythemia Vera (PV) and Primary Myelofibrosis (PMF), the clonal nature is reflected by a gain-of-function mutation in the JAK2 gene, which was identified using a functional approach [1]. The JAK2V617F mutation is specific for myeloid neoplasms and is present in approximately 95% of patients with PV and in 50–60% of patients with ET and PMF, respectively [2]. Moreover, JAK2 exon 12 mutations are present in rare cases of JAK2V617F-negative PV as well as MPL mutations in JAK2V617F-negative ET and PMF. In addition, using a whole genome sequencing approach, Klampfl *et al*. *and Nagalia et al* identified calreticulin (CALR) mutations in the majority of ET and PMF patients that are negative for JAK2 or MPL alterations [3,4]. Furthermore, somatic mutations in other genes, such as TET2, DNMT3A, ASXL1, EZH2, IDH1/2, U2AF1, SF3B1, SRSF2, CBL, NF-E2, SH2B3 (LNK), CHEK2 [3], and SOCS 1, 2 and 3, IKZF, SETBP1, among others, have been found in all stages of MPN [5–9]. Furthermore, frequent CSF3R mutations were found in chronic neutrophilic leukemia and atypical CML [10]. Additionally, in eosinophilic MPN, fusion proteins with constitutive tyrosine kinase activity involving PDGFRα, PDGFRβ, and FGFR1 have been described. In systemic mastocytosis (SM), a gain-of-function mutation in the tyrosine kinase receptor (KIT D816V) contributes to cell-autonomous proliferation of atypical mast cells.

Beyond additional diagnostic markers, NGS provides quantitative information on sequence abnormalities, which can be used to estimate the clone size and thus allows to identify potential driver mutations in a given patient. Also, NGS enables the detection of clonal evolution over the course of the disease or during treatment[9]. Moreover, NGS permits the distinction of homozygous versus heterozygous mutations as well as cis- or trans-located compound mutations. Finally, by identifying multiple mutations and even low-level mutations, NGS has revealed potential prognostic impact of various mutations, as shown for CML [11], myelofibrosis [12], and systemic mastocytosis [13].

However, despite these developments, multiple questions remain regarding (i) the best method to screen for these mutations, (ii) the distinction between relevant and irrelevant mutations as well as the precise pathogenetic and phenotypic role of the known aberrations for each of the MPN subtypes, (iii) changes of subclones during treatment and (iv) the prognostic role of single vs. combined mutations (i.e. TP53 mutation alone or together with an IDH1 mutation). In particular, the identification of factors contributing to disease initiation and progression, as well as the respective influence on the clinical disease course is needed to optimize the diagnosis, prognostic scoring and therapeutic approaches, and valid cutoff values are needed when describing infrequent mutations. Here, we used next generation amplicon sequencing to investigate the mutational landscape of MPN subtypes as well as its correlation with clinical characteristics of the patients, showing that the present approach is feasible for future personalized diagnosis and treatment approaches.

## Materials and Methods

### Cell lines

Cell lines included K562 (CML in blast crisis), HEL (erythroleukemia), HMC-1.2 (mast cell leukemia), SUP-B15 (Bcr-Abl positive B cell precursor leukemia), HL60 (acute myeloid leukemia), U937 (histiocytic lymphoma), KCL-22 (CML in blast crisis) cells (http://www.dsmz.de/catalogues/catalogue-human-and-animal-cell-lines.html). Routinely, cell lines were cultured in RPMI with 10% fetal bovine serum. SUP-B15 cells were cultured in IMDM with 20% fetal bovine serum.

### Primary cell samples

Samples from 64 patients with MPN (19 MF [10 PMF, 9 post-PV/ET-MF] 17 PV, 10 ET, 11 CML, 5 HES, and 2 SM), three patients with reactive erythrocytosis and two anonymized healthy controls were used for sequencing (Table 1). The diagnostic criteria used for the establishment of the MPN diagnosis were defined according to WHO 2008 classification of myeloid neoplasms. After written informed consent, peripheral blood and bone marrow samples were taken from the patients. Blood cells were subjected to erythrocyte lysis and cells were stored at -20°C until further use. The study was approved by the local ethics review board (ethics committee of the Faculty of Medicine of the RWTH Aachen) (EK206/09, EK127/12, EK061/14). One patient died before signing informed consent and the analysis of this sample was approved posthumously (EK061/14) and ethics committee waived the need for consent. Written consent was obtained from all other patients before sample analysis. Patients specifically consented to publication of anonymized clinical data as presented in this publication.

**Table 1:** Patient characteristics.

Patient no.	Disease entity	Origin of sample	BCR-ABL status	JAK2V617F (allele burden)	Spleen size (below costal margin)
1	CML-AP	bm	positive	NA	enlarged 18cm
2	CML-BC	pb	positive	NA	not enlarged
3	CML-AP	bm	positive	NA	not enlarged
4	CML-CP	bm	positive	negative	not enlarged
5	CML-CP	pb	positive	NA	enlarged
6	CML-CP	pb	positive	negative	not enlarged
7	CML-CP	bm	positive	NA	not enlarged
8	CML-CP	bm	positive	NA	not enlarged
9	CML-CP	pb	positive	NA	enlarged 10cm
10	CML-CP	pb	positive	NA	NA
11	ET	pb	negative	V617F (64%)	not enlarged
12	ET	pb	negative	negative	not enlarged
14	ET	pb	negative	negative	NA
15	ET	pb	negative	negative	not enlarged
16	ET	bm	negative	negative	not enlarged
17	ET	pb	negative	V617F (35%)	not enlarged
18	ET	pb	negative	negative	not enlarged
19	ET	pb	NA	negative	not enlarged
20	ET	pb	negative	V617F (16%)	not enlarged
21	ET	pb	negative	V617F (18%)	not enlarged
22	HES	bm	negative	negative	not enlarged
23	HES	bm	NA	negative	not enlarged
24	HES	pb	negative	negative	not enlarged
25	HES	pb	negative	V617F (80%)	not enlarged
26	HES	pb	NA	NA	NA
27	MF post ET	pb	negative	95%	enlarged 20cm
28	MF post PV	pb	negative	71%	enlarged 10cm
29	MF post PV	pb	negative	93%	enlarged 12cm
30	MF post PV	pb	NA	99%	enlarged 24cm
31	MF post PV	pb	negative	93%	enlarged 9cm
32	MF post PV	pb	negative	99%	enlarged 30cm
33	MF post PV	pb	negative	52%	enlarged 15cm
34	MF post PV	pb	NA	99%	enlarged 18cm
36	pMF	pb	negative	46%	not enlarged
37	MF post PV	pb	negative	82%	enlarged 12cm
38	pMF	pb	negative	31%	enlarged 15cm
39	pMF	pb	negative	65%	enlarged 10cm
40	pMF	pb	negative	negative	enlarged 8cm
41	pMF	bm	NA	28%	enlarged 10cm
42	pMF	pb	negative	negative	enlarged
43	pMF	pb	negative	10%	not enlarged
44	pMF	pb	negative	87%	enlarged 4cm
45	pMF	pb	negative	41%	enlarged 5cm
46	pMF	pb	negative	negative	NA
47	CML+ PV	pb	positive	54%	not enlarged
48	PV	pb	negative	28%	not enlarged
49	PV	pb	negative	15%	not enlarged
50	PV	pb	NA	84%	not enlarged
51	PV	pb	NA	37%	enlarged 1–2cm
52	PV	pb	NA	32%	not enlarged
53	PV	pb	NA	93%	enlarged 6cm
54	PV + systemic mastocytosis	pb	NA	29%	not enlarged
55	PV	pb	negative	88%	enlarged 10cm
56	PV	pb	negative	30%	not enlarged
57	PV	pb	negative	41%	enlarged 2cm
58	PV	pb	negative	76%	not enlarged
59	PV	pb	negative	96%	NA
60	PV	pb	NA	19%	not enlarged
61	PV	pb	negative	27%	not enlarged
62	PV	pb	negative	42%	not enlarged
63	PV	pb	negative	23%	enlarged 11cm
64	RE	pb	negative	negative	not enlarged
65	RE	bm	negative	negative	not enlarged
66	RE	pb	negative	negative	not enlarged
67	SM	bm	NA	NA	enlarged
68	SM	pb	negative	negative	not enlarged
69	CML-BC/Ph+ ALL	pb	positive	n.d.	NA

Abbrevations: CML = chronic myeloid leukemia, CP = chronic phase, AP = accelerated phase, BC = blast crisis; ET = essential thrombocythemia; PV = polycythemia vera; HES = hypereosinophilic syndrome; MF = myelofibrosis, ALL = acute lymphoblastic leukemia, bm = bone marrow; pb = peripheral blood. If JAK2V617F positive, the allele burden of the c.1849G>T mutation (which leads to V617F) detected by NGS is shown in percent of total JAK2 sequences

### Isolation of genomic DNA

Genomic DNA (gDNA) was extracted by use of the Qiagen QIAamp DNA Blood Mini Kit according to manufacturer’s protocol. DNA quantification was done using the Qubit 2.0 Fluorometer as well as the Nanodrop 2000c Spectrophotometer. For subsequent sequencing, the gDNA concentration of all samples was adjusted to 50ng/μl.

### Next-generation sequencing and analysis of sequencing results

A multiplex-PCR approach (Truseq Amplicon Cancer Panel, Illumina) of 212 amplicons covering genomic mutational hotspots in 48 cancer-related genes was used to identify mutations in a cohort of patients with MPN (S4 Table). The manifest file with distinct data of all primers/amplicons can be downloaded at http://support.illumina.com/downloads/truseq_amplicon_-_cancer_panel_manifest_file.html. 5μl (250ng) of gDNA of each sample was prepared according to the TruSeq sample preparation guide. Data were analyzed using MiSeq reporter (Illumina), BaseSpace online analysis tool (Illumina) and SeqPilot software. To avoid scoring of mutants that were detected as a result of unfaithful PCR amplification or sequencing, a sequence variant was only further analyzed when the following conditions were met: (1) the absolute coverage at the variant site was ≥50 (2) the variant was detected in at least 10 (absolute) reads, and (3) the variant was detected in 5% of all reads at the variant site. For potential pathogenic effects of new detected variants, additional analysis was performed using MutationTaster software (http://www.mutationtaster.org) and PolyPhen-2 software (http://genetics.bwh.harvard.edu/pph2/bgi.shtml).

### Statistics

Statistical analysis was performed using a two-sided T-Test (using IBM SPSS Statistics 20 software). Differences in the coverage of several genes/amplicons were calculated using non-parametric tests (Kruskal-Wallis Test).

## Results

### Amplicon-based NGS for detection of MPN mutations

**Fig 1:**
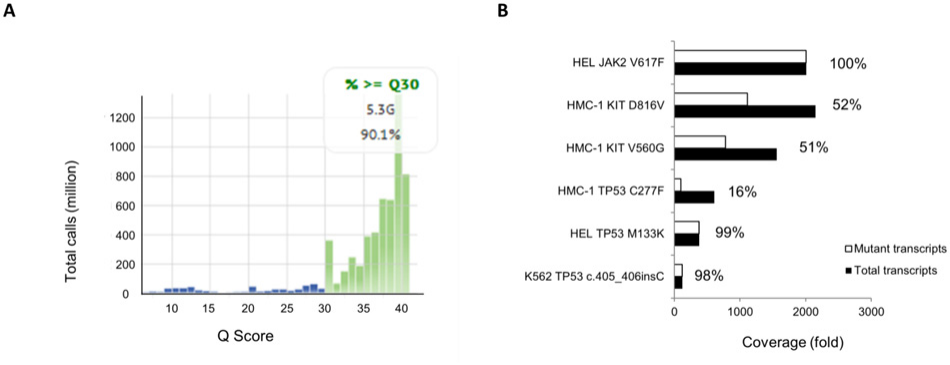
Quality control of next generation sequencing. (A) Quality of sequencing as assessed by Phred Quality Score (Q-Score) is shown for the second sequencing run, yielding 5.3 GB of data. 90.1% of all bases were called with a Q-Score of 30 or better (indicating that the probability of an incorrect base call was 1:1000 or less). (B) Coverage of wild-type and mutant reads from known mutations in HEL, HMC-1, and K562 cell lines is shown, as analyzed by SeqPilot Software. Percentages display the fraction of mutant reads per total reads in each case, with values close to 100% indicating homozygous and values close to 50% indicating heterozygous mutations.

The coverage and sensitivity of amplicon-based sequencing are dependent on the number of samples that are analyzed within the same approach, with both parameters being adversely affected by too few or too many samples used for one analysis. Therefore, to optimize both parameters, we performed two separate runs using 48 and 40 samples each from patients with MPN or with reactive erythrocytosis, healthy controls, and 7 cell lines. Samples from some patients were analyzed in both runs and some of the samples included quality controls. High quality of both runs was confirmed by Q30 values >90% (Fig 1A). 151 bidirectional cycles were performed, yielding between 2 and 6 Gigabases of sequencing data.

**Fig 2:**
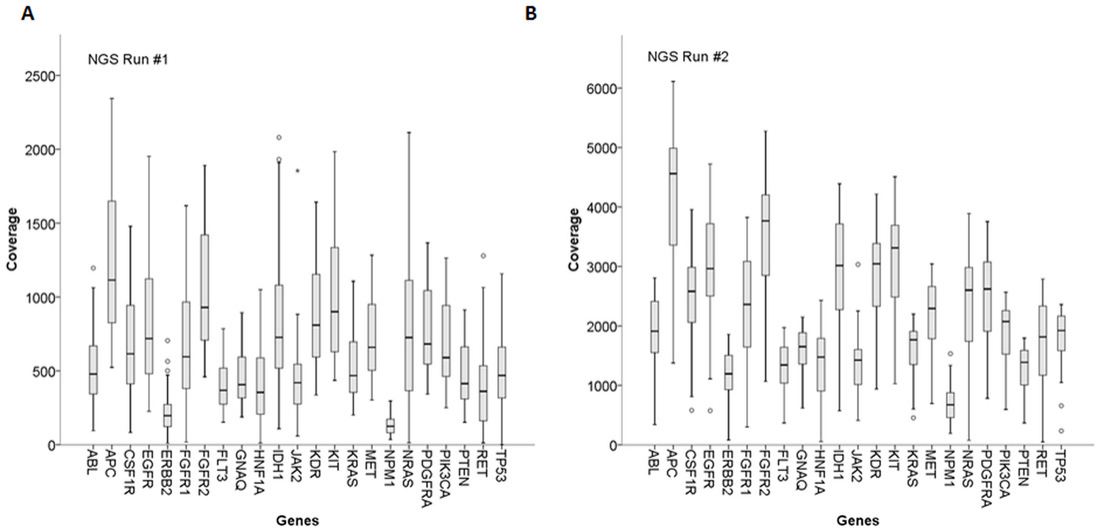
Mean coverage of selected amplicons. **(A) First run: The mean coverage (y-axis) of all tested samples of the first run of selected amplicons (gene name shown on x-axis).** (B) shows the same as A) for the second run. The difference of the coverages among the different amplicons was tested using a Kruskal-Wallis nonparametric test.

Overall median coverage in the first run was 546 in those genes that were found to be mutated in at least on patient, with the coverage varying between 125 and 1115 among different genes (Fig 2). More specifically, Kruskal-Wallis statistical testing showed NPM1 and ERBB2 coverage to be lowest and significantly different from all of the other gene coverages (125 and 197, respectively), but nevertheless sufficient for mutational analysis. A second group of gene primers (RET, HNF1A, FLT3) also produced significantly lower coverages than the other gene primers, while APC gene coverage was highest among all tested genes (Fig 2).

In the second run, median coverage was 1953 reads and was thus 3.6-fold higher than in the first run. Again, NPM1 and ERBB2 coverages were lowest (674 and 1195, resp.) and that of APC was highest (4561), and the relative coverages were very similar to those of the first run, suggesting high reproducibility of PCR conditions for each amplicon, but different sequencing efficiency in the two sequencing runs. Five of the 212 primer combinations did not result in sequence output suggesting that no amplicons had been generated (S2 Table) and several sequences may have reflected pseudogene amplification (S3 Table).

Healthy donor and reactive erythrocytosis samples showed several known frequent single nucleotide polymorphisms (SNPs) but no known pathogenic mutation and the amount of SNPs found in the 48 genes that were sequenced ranged from 2 to 6 in the different samples (S1 Table). Sequencing of the CML-BC cell line K562 confirmed the presence of a previously described TP53 frameshift mutation (c.405_406insC; in 98% of transcripts). Homozygous JAK2V617F was demonstrated by detection of c.1849G>T mutation in 100% of sequencing reads in the erythroblast cell line HEL (Fig 1B). Moreover, we could confirm a recent report[14] showing that HEL cells harbor a TP53 M133K (99%) mutation that is located in the DNA-binding domain of p53. Further to the two heterozygous KIT mutations V560G (51%) and D816V(52%) that are known to be present in the mastocytosis cell line HMC-1.2, we found an acquired TP53 C277F mutation in 16% of sequencing reads, suggesting that TP53 mutations might develop during passages of KIT mutated cells. Cell lines derived from patients with CML (KCL-22), ALL (SUP-B15), AML (HL60), and histiocytic lymphoma (U937) showed no abnormality in the tested gene set. The number of SNPs was comparable to the detected SNPs in patients´ samples.

### JAK2 V617F allele burden and single nucleotide variations in MPN

**Fig 3:**
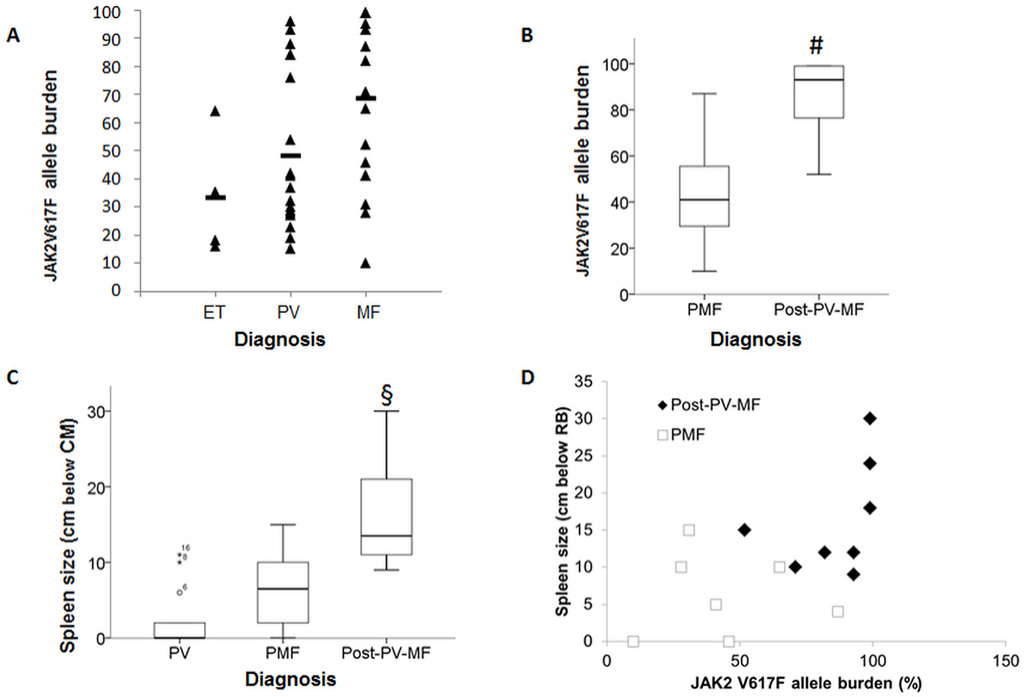
JAK2 V617F allele burden differs in the indicated entities. (A) Analysis of the JAK2 allele burden of JAK2 V617F positive MPN samples: ET n = 4, PV n = 17, MF n = 16. Black bars show the mean allele burden. The allele burden showed significant differences even in a small subgroup of MPN samples (see text). (B) Addressing only the MF samples (n = 16), pMF samples (7/16) had a significant lower allele burden compared to Post-PV-MF samples (8/16); # p<0–01 (T-Test), the one Post-ET-MF sample was not included in this analysis. (C) The extent of splenomegaly of the different MPN (ET, PV, MF) was different in indicated entities (presented in cm below costal margin), e.g. spleen size of Post-PV-MF was largest vs. PMF and PV (p<0.01, One-way ANOVA and LSD testing). (D) Relationship between JAK2 allele burden and spleen size. A higher allele burden was associated with a larger spleen in post-PV-MF (R^2^ = 0,1841) but not in PMF (R^2^ = 0,00002). Abbrevations in this figure are the same as used in the text.

The JAK2 c.1849G>T (V617F) mutation was detected in all PV (17 of 17), 4 of 10 ET, 7 out of 10 PMF, 1 out of 1 post-ET-MF, and in 8 out of 8 post-PV-MF patients. The JAK2 c.1849G>T (V617F) allele burden was highest in MF samples (64+/-28%) followed by PV (43+/-28%) and ET (28+/-24%) (Fig 3A). The allele burden was significantly higher in MF than ET (p = 0.026 by LSD testing) and PV (p = 0.039; LSD testing), while there was no significant difference between PV and ET (p = 0.33; LSD testing). Among MF samples only, a significantly higher JAK2V617F allele burden was detected in post-PV-MF (mean = 86+/-17%) than in PMF samples (mean = 44+/-25%) (p = 0.009; T test) (Fig 3B). The single post-ET-MF sample was not included in this analysis but showed a high mutant allele burden of 95%. Splenomegaly at the time of NGS analysis was evident in 5 of 15 evaluable PV, 6 of 8 evaluable PMF, and 8 of 8 post-PV-MF patients with median spleen sizes of 2.0+/-3.8cm, 6.5+/-5.2cm, and 16.3+/-7.4cm, resp., (p<0.01 for Post-PV-MF vs. PV and PMF, LSD testing) below the costal margin (Fig 3C). There was a significant correlation between spleen size and JAK2 mutant allele burden (p = 0.027 by Pearson´s testing, Fig 3D)

For JAK2V617F positive PMF (sample 36, allele burden 46%) an additional IDH1 c.395G>A variant was detected (24% of reads, leading to R132H amino acid exchange). This patient had been diagnosed with PMF but showed an unusual disease phenotype, including basophilia (up to 26% basophils in the peripheral blood), hyperhistaminemia, and daily flush symptoms of the skin, which only lasted for a few seconds. Systemic mastocytosis was repeatedly excluded by morphology and by the absence of the KIT D816V mutation, and serum tryptase levels were normal. Although ruxolitinib treatment induced a hematologic response in this patient, elevated basophils and flush symptoms persisted and were treated symptomatically with desloratadin and montelukast.

A SMO c.1004T>C (L335P) variant (11% of reads) was found in a second post-PV-MF patient (in sample 43) with a JAK2V617F allele burden of 99%, an enlarged spleen (18 cm below the costal margin) and grade 2 MF. The SMO c.1004T>C variant leads to an amino acid exchange (leucine→proline, both nonpolar and hydrophobic amino acids) in codon 335, the latter of which is located in the transmembrane domain of the smoothened homolog.

A third JAK2V617F positive (allele burden 31%) post-PV-MF patient (no. 38) with splenomegaly (15cm) and grade 3 MF showed a c.764A>G (E255G) ABL variant in 10% of the sequencing reads.

Moreover, we detected two ATM variants in three JAK2V617F positive patients with PV or post-PV-MF but not in healthy controls or other entities. The variant ATM c.2572T>C (F858L, dbSNP: rs1800056) was homozygously present in one patient with PV (JAK2V617F 19%, spleen not enlarged, no significant fibrosis) and heterozygous in one patient with post-PV-MF (JAKV617F 52%, 15cm enlarged spleen, MF). A further heterozygous ATM variant (c.5071A>T (S1691C)) was detected in another post-PV-MF patient (JAK2V617F 93%, 12cm spleen grade 2 MF).

Furthermore, one of the three JAK2V617F negative MF patients (sample 42) showed a previously described c.35G>T (G12V) NRAS variant (13% of reads).

### Identification of mutations in PV

Further to the homozygous ATM variant in the one PV patient described above, we detected one CSF1R variant (17% of all reads, c.2804G>A (S935N)) in this cohort (JAK2V617F 41%, spleen size of 2cm below the costal margin, MF grade 0) predicted as polymorphism by mutation taster and polyphen-2 software.

Since LNK mutations were described in MPN but escaped detection by the Truseq Amplicon Cancer Panel, we performed Sanger sequencing for the hotspot region LNK exon 2 for all samples. In one of the JAK2 V617F-positive PV patients, we found one LNK (E208Q) mutation which has previously been described only in a JAK2V617F negative MPN patient[15]. The patient in our study did not respond to hydroxyurea and was one out of five PV patients with splenomegaly (PV total n = 16). The spleen size was 10 cm vs 6+/-5 cm and grade 1 MF was evident. We did not detect any other LNK exon 2 mutation in the entire cohort of our 63 patients.

Moreover, the analysis included one patient that had been diagnosed with Bcr-Abl positive CML and JAK2 V617F positive PV. Initial imatinib therapy had failed to induce cytogenetic response, and nilotinib therapy was implemented, which was stopped four years later due to peripheral artery occlusive disease. By that time, a JAK2V617F mutation was detected (54%) and we included this sample for NGS which revealed an additional IDH1 c.374T>G (V125G) mutation (9% of all reads).

### Sequence variants in patients with essential thombocythemia, hypereosinophilic syndrome, or systemic mastocytosis

We included 10 essential thrombocythemia (ET), 5 hypereosinophilic syndrome (HES), and 2 systemic mactocytosis (SM) patients in our analysis. Within the ET patient cohort, 4 out of 10 were positive for JAK2V617F. Among these 4 patients, we detected one patient with a MET c.3029C>T (T1010I) variant (54% of all reads, JAK2V617F 64%); the spleen size was normal, and the patient is currently treated with hydroxyurea. We did detect any further variation within the analyzed gene set of the remaining ET patients. The same MET variant detected in the ET patient was also present in 1 out of 5 HES patients (54% of reads). We detected one KRAS variant in one out of two SM patients 16 months after diagnosis. This c.436G>A mutation (32%) was located in exon 4 of the KRAS gene and results in an A146T amino acid exchange. By the time of sequencing, this patient had progressed to SM-AHNMD with an enlarged spleen (23cm below costal arch), grade 2 MF, and an elevated tryptase level of >200 ng/ml.

### Detection of mutations in CML

**Fig 4:**
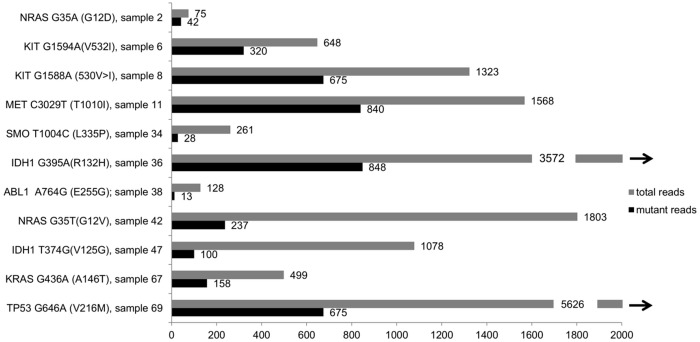
Coverage of detected single nucleotide variations (SNVs). The y axis shows the detected variant with the base exchange and the resulting amino acid exchange. Furthermore, the sample number is shown. Sample details and clinical data can be extracted from the corresponding sample number in Table 1 (Patient characteristics). The x axis shows the absolute allele burden of mutant reads (grey) and total reads (black) at each SNV site, with the exact numbers shown at the end of the bar. The maximum of x axis was limited to 2000. The black arrows demonstrate that the coverage of these two samples was higher than 2000 (exact numbers given).

Further to the above described CML/PV patient with Bcr-Abl and JAK2V617F, we included eleven CML samples in our analyses. By the time of sample collection, seven of those were in CML-CP, two in CML-AP, and further two in CML-BC (one of them Ph+ALL/CML-lyBC). Three of the eleven CML patients showed subclones with sequence variants in the HNF1A gene (Fig 4), including a S304P variant in one CML-CP and one CML-AP sample (both 9% of all reads) and one 872delC mutation (6%) in a further CML-CP patient, but due to low allele burden we were not able to validate this variants by conventional Sanger Sequencing.

Moreover, we detected two previously described KIT variants, causing AA exchanges in the transmembrane domain, V530I (51% patient 8) and V532I (49%, polymorphism rs3822214) (sample 6). The KIT V530I mutation has previously been described in CBF-AML. However, Sanger sequencing using MACS-purified CD3 cells revealed the mutation being a germline variant in this patient (S1 Fig).

**Fig 5:**
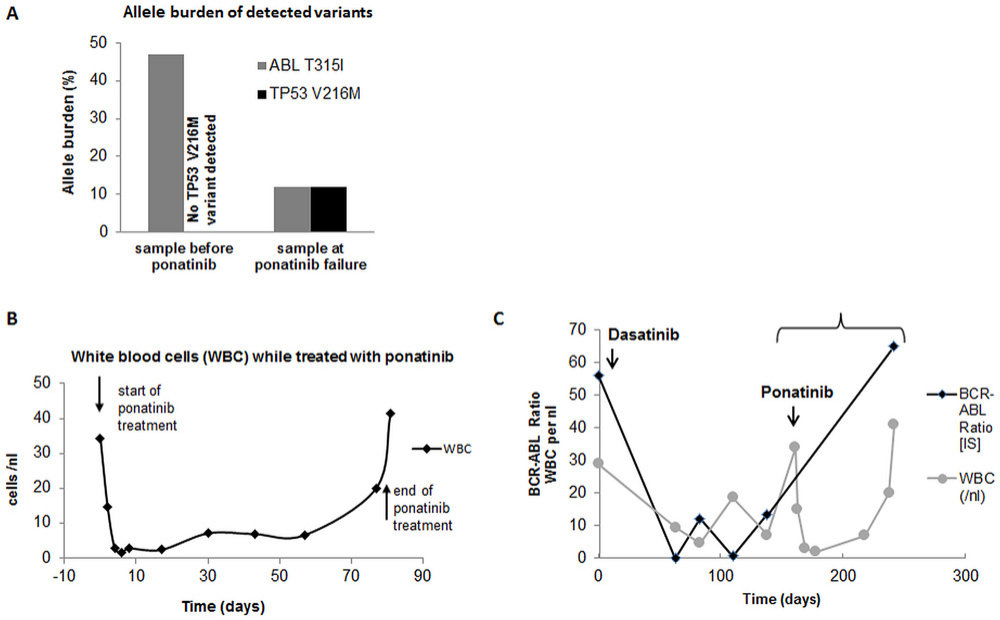
Follow up analysis (sample 69). **(A) Allele burden of ABL T315I and TP53 V216M variants in a CML-BC/Ph+ ALL sample.** The left bar shows the ABL T315I allele burden after dasatinib failure and before start of ponatinib, the V216M variant was not detected at this time point. Right bars show allele burdens of ABL T315I and TP53 V216M at the time of ponatinib failure. (B) The white blood cell count (WBC) from the same time period is presented. As shown, leukocyte count decreased when ponatinib treatment started and increased at the time of ponatinib failure. The two black arrows indicate the time points when the allele burden was analyzed (see Fig 5A). (C) BCR-ABL ratio (black) and WBC (grey) of the same patient over a longer time period from start of dasatinib treatment to ponatinib failure. The parenthesis indicates the same time period shown in Fig 5B.

In another patient with Ph+-ALL/CML-lyBC (patient 69) who had a known ABL c.944C>T (T315I) mutation, we confirmed this mutation (47% of reads mutated, leading to T315I AA exchange). By that time, no other mutation was detected in the analyzed gene set. Upon initial dasatinib and methotrexate treatment, Bcr-Abl major levels were reduced by 3 logs but subsequently increased again. Upon relapse, we performed a second analysis. Interestingly, in this sample, we now detected a TP53 c.646G>A (V216M) variant (12% of reads), which was acquired during treatment with ponatinib. Also, the patient had now acquired an E279K ABL mutation in 4% of total sequences as confirmed by Sanger sequencing (detection of 30% of all Bcr-Abl fusion transcripts harboring the E279K mutation). The ABL c.944C>T allele burden decreased to 12% of all reads and the patient became ponatinib-resistant shortly after the sample was taken (Fig 5A–5C).

A second patient with CML-BC was analyzed: this 35 year-old patient with CML (first diagnosed 11 years prior to presentation in our hospital) had undergone allogeneic stem cell transplantation after imatinib failure (loss of molecular response after two years of imatinib treatment). After complete cytogenetic response (nested PCR was still positive), CML recurred seven years later, and he was treated with nilotinib and subsequently dasatinib, After treatment failure, a second stem cell transplantation followed one year later. An early relapse a year later was treated with ponatinib with no sufficient response, and the patient died shortly thereafter with persisting blast crisis. No mutation in the Bcr-Abl fusion gene had been detected by conventional sequencing. Analysis of a sample of this patient taken in before the initiation of ponatinib treatment confirmed the absence of an ABL mutation but revealed a NRAS c.35G>A (G12D) variant in 53% of all reads. This mutation is well known for its effects on proliferation and its association with AML and MPN [16,17], suggesting that this variant might have been involved in the TKI resistance of this patient.

## Discussion

The NGS approach reported here produced interesting results, which could not have been gathered by conventional Sanger sequencing. While the mutational pattern, as previously described for MPNs, was reproduced, some additional novel SNVs and potential novel disease associations were uncovered. Amplicon-sequencing-based NGS allowed simultaneous analysis of a wealth of MPN-associated genes at diagnosis as well as during treatment, provided a quantitative measurement of the mutant allele burden, distinguished between homozygous and heterozygous mutations as well as compound mutations in cis or trans location. Furthermore, it enabled us to analyze the role of genes previously described primarily in solid tumors. There are several caveats in using commercial amplicon kits, including a certain variance in amplicon coverage and the fact that the BaseSpace software does not discriminate between normal genes and pseudogenes.

We used well-known hematopoietic cell lines with known mutations to validate our NGS approach. In fact, all known mutations were detected in our experiments. Interestingly, we found one additional mutation, the TP53 “DNA contact mutant” C277F found in 16% of reads in the mastocytosis cell line HMC1.2. This mutation has previously been shown to exert impaired suppression of colony formation of transfected Saos-2 cells [18,19], thus suggesting a role in tumor progression. Currently, at least twenty C277F mutants have been described in maligant tumor specimens of the lung, hematopoietic system, esophagus, breast, and skin (http://cancer.sanger.ac.uk/cosmic/mutation/overview?id=10749), including a case of therapy-resistant chronic lymphocytic leukemia [20] and one with follicular lymphoma [21]. Finally, our NGS data show that cell lines may be less homogeneous than previously thought, not only phenotypically, but also genetically. None of our negative control samples (healthy controls, reactive erythrocytosis) showed any evidence of tumor-associated mutations, again validating our NGS approach.

JAK2 V617F mutations occur in over 95% patients with PV and approximately 50% of patients with ET or PMF, and these entities are associated with different allele burden levels [22–25], with ET harboring the lowest JAK2 allele burden as compared to PV and PMF [26,27]. The allele burden is similar in peripheral blood and bone marrow [28], and it remains stable over several years [29]. Most of those studies used techniques other than NGS to measure the allele burden (e.g. qPCR, Pyrosequencing etc. [30]). But with NGS, it is possible to separate heterozygous from homozygous cases and potentially provide a more accurate tool for MRD disease monitoring [31]. In our study, JAK2 mutations were with the lowest JAK2 allele burden in ET samples and highest in secondary MF samples, which is well in accordance with previous findings [2].

When screening for additional mutations, a LNK E208Q mutation was detected in a patient with JAK2V617F positive PV, who had been refractory to hydroxyurea at the time of sampling. It is tempting to speculate that the LNK mutation provided an additional means of enhancing the JAK2-STAT pathway, potentially rendering the PV cells more resistant to cytoreductive therapy. To our knowledge, this is the first case of PV with concomitant JAK2V617F and LNK mutations. A subsequent MPN-specific panel will have to be included in the analysis of LNK mutations as well as other important mutations (i.e. calreticulin).

IDH1 mutations have been described in MPNs [32] and AML [33]. In our series, we detected one IDH1 c.374T>G (V125G) variant, and one c.395G>A (R132H) variant, but the relevance and germline state remains unclear. However, the substitution of arginine to histidine at position 132 enables the enzyme to catalyze the reduction of alpha-ketoglutarate to R(-)-2-hydroxyglutarate (2HG) [34], with the 2HG level being discussed to be a predictor for outcome in AML [35], possibly by augmenting the differentiation block [36] and the ROS level [37].

Another individual variant in SMO was revealed in our analysis with the limitation of the unknown germline state. Smoothened is part of the sonic hedgehog pathway and recent studies show activity of a Smoothened antibody (Vismodegib) in basal cell carcinoma [38]. However, this sequence variant has not been described to be associated with tumors, and its pathogenic role in MPN remains to be elucidated (i.e. in the current clinical phase 2 trial of LDE225 and ruxolitinib in patients with MF [trial identifier NCT01787552])

Furthermore, one of the three JAK2 negative MF patients (sample 42) showed a previously described c.35G>T (G12V) NRAS variant (13% of reads). Since this mutation is known from many cancers, including PMF [39], it is possible that it plays a role in the pathogenesis of MF in this patient.

Another MF sample (no. 38) showed a c.764A>G (E255G) ABL variant in 10% of the reads. While it is unlikely that this mutation plays a pathogenic role, appearance of this variant may indicate genomic instability in this patient.

Mutational analysis is standard clinical practice in patients with CML, who experience secondary resistance to tyrosine kinase inhibitor (TKI) treatment [40]. Additionally, the presence of specific ABL mutations, such as the T315I mutation, already has a major impact on the choice of the individual TKI to be used. We suggest here that detection of low-level mutations by NGS should provide further important information when selecting one of the second- or third-generation TKIs [11]. So far it is not known, which threshold is needed for the low-level mutations and whether the retrospective data can be reproduced in prospective trials.

One of our patients with CML-BC/Ph+ ALL (no. 69) acquired an additional TP53 (V216M)variant during ponatinib treatment (in 12% reads), a mutation known to be associated with breast cancer [41,42]. Comparing the first and the second sample of this patient, a decrease of the ABL allele burden from 47% (before ponatinib) to 12% was detected, suggesting that the T315I and V216M mutants were found in the same subclone. Shortly after the second sample had been taken, the patient became ponatinib resistant, and because TP53 is well known to be important for the response to TKI, this TP53 variant may have be one factor leading to ponatinib resistance in this patient. This example again highlights the fact that even mutations in the 10–20% range may be of clinical importance when the cells harboring these mutations are exposed to external stressors such as TKI treatment.

As described above, one oncogenic KIT variant was found in the transmembrane domain of the KIT protein, namely V530I. The V530I mutant from patient 8 is known to show IL3 independent growth in FDC-P1 cells [43] and seems to have an effect on imatinib sensitivity in desmoid tumors [44]. Therefore, we identified the mutation as a germline variant. Nevertheless, it cannot be excluded that this variant might have conferred an advantage to the disease-causing clone.

Both patients with systemic mastocytosis had previously tested positive for the presence of a KIT mutation by allele-specific PCR (KIT D816V). Interestingly, in both patients, NGS failed to detect any KIT mutation, suggesting that the KIT positive cells comprised only a minor portion of the malignant clone. However, in one of the two patients who also suffered from an associated clonal hematological non-mast cell lineage disease, NGS detected a KRAS exon 4 mutation. KRAS is part of the RAF-KRAS-MEK pathway, which finally influences ERK that plays an important role in cell differentiation and proliferation [45,46]. The detected c.146A>T variant is known from colorectal cancer and leads to EGFR-therapy resistance in this entity [47–49]. Furthermore, this mutation was described in AML and CMML [50], and biochemical and functional assays showed oncogenic properties of this mutation [50]. The course of disease in this patient has been rather complicated with rapid progressions following each of the applied treatments, such as hydroxyurea and cladribine. This case illustrates the finding that an increasing number of mutations further to the KIT D816V mutation are associated with decreased survival [12].

In conclusion, implementation of amplicon based NGS in diagnosis and molecular classification of MPNs represents an important advancement, as it allows rapid parallel analysis of multiple mutations, detection of clonal hierarchies and clonal evolution as well as molecular monitoring during cytoreductive treatment and targeted therapy of MPNs.

## Supporting Information

S1 Table Single nucleotide variations. Detected single nucleotide variations (SNV) or single nucleotide polymorphisms (SNP) listed in dbSNP 137 data base listed by sample (first column: sample no., compare Table 1 patient characteristics)
(DOCX)

S2 Table Amplicon regions with no amplikcon product. List of regions which were analyzed by base space software (illumina) which showed amplicon regions with no amplification product.
(DOCX)

S3 Table Pseudogene regions. List of primers with amplification of pseudogene regions. First column shows primer name (basespace, illumina), second column shows pseudogene location (chromosome)
(DOCX)

S4 Table List of genes included in the TruSeq cancer amplicon panel. (DOCX)

S5 Table Primer for sanger sequencing. List of primers used for validation of detected variants by conventional sanger sequencing (upper part) and allele specific PCR (lower part, according to Jones *et al*., BLOOD, 2005)
(DOCX)

S1 Fig Germline variant V530I. The previously described c-kit V530I SNP was present in total PB-derived as well as CD3+ selected T cells from a patient with CML. CD3 MACS-sorted cells (left) and CD3-depleted Cells (right) were stained as control using CD3-FITC antibody. Sanger sequencing was performed using CD3 pos T-cell population (lower panel)
(TIF)

S2 Fig Validation of the MET c.3029C>T variant by Sanger sequencing in sample 11. (TIF)

S3 Fig Validation of the IDH1c.395G>A variant by Sanger sequencing in sample 36. (TIF)

S4 Fig Validation of the CSFR1 c.2804G>A variant by Sanger sequencing in sample 57 (TIF)

S1 File Supplement Methods. Additional methods not mentioned in the methods part of the manuscript.
(DOCX)
