# Supplementary material for: Dissecting Genomic Aberrations in Myeloproliferative Neoplasms by Multiplex-PCR and Next Generation Sequencing
Source: PLoS One. 2015 Apr 20;10(4):e0123476. doi: 10.1371/journal.pone.0123476 (PMC4404337; doi:10.1371/journal.pone.0123476)
Supplement: S1 Table — Detected single nucleotide variations (SNV) or single nucleotide polymorphisms (SNP) listed in dbSNP 137 data base listed by sample (first column: sample no., compare Table 1 patient characteristics) (DOCX) [file pone.0123476.s001.docx]

**Supplement 1 (Table)**:

|  | Gene | | | | | | | | |
| --- | --- | --- | --- | --- | --- | --- | --- | --- | --- |
| **no.** | APC | ATM | CDH1 | EGFR | KDR | KIT | MET | PDGFRA | TP53 |
| 1 | rs41115 |  |  | rs1050171 | rs1870377 |  |  | rs1873778 |  |
| 2 | rs41115 |  |  | rs1050171 |  |  |  | rs1873778 |  |
| 3 | rs41115 |  |  | rs1050171 |  |  |  | rs1873778 |  |
| 4 | rs41115 |  |  | rs1050171 |  |  |  | rs1873778 |  |
| 5 | rs41115 |  |  | rs1050171 |  |  |  | rs1873778 |  |
| 6 | rs41115 |  |  | rs1050171 |  |  |  | rs1873778 |  |
| 7 |  |  |  | rs1050171 |  |  |  | rs1873778 | rs1042522 |
| 8 | rs41115; rs1801166 |  |  | rs1050171 |  |  |  | rs1873778 |  |
| 9 | rs41115 |  |  |  |  |  |  | rs1873778 |  |
| 10 | rs41115 |  |  |  |  |  |  | rs1873778 | rs1042522 |
| 11 | rs41115 |  |  | rs1050171 |  |  |  | rs1873778 |  |
| 12 | rs41115 |  |  | rs1050171 |  |  |  | rs1873778 |  |
| 14 | rs41115 |  |  | rs1050171 |  |  |  | rs1873778 |  |
| 15 | rs41115 |  |  | rs1050171 |  | rs3822214 |  | rs1873778 |  |
| 16 | rs41115 |  |  | rs1050171 |  |  |  | rs1873778 |  |
| 17 | rs41115 |  |  | rs1050171 |  | rs3822214 |  | rs1873778 | rs1042522 |
| 18 | rs41115 |  |  | rs1050171 |  |  |  | rs1873778 | rs1042522 |
| 19 | rs41115 |  |  | rs1050171 |  |  |  | rs1873778 |  |
| 20 | rs41115 |  |  | rs1050171 |  |  |  | rs1873778 |  |
| 21 |  |  |  | rs1050171 |  |  |  | rs1873778 |  |
| 22 | rs41115 |  |  | rs1050171 |  |  |  | rs1873778 | rs1042522 |
| 23 | rs41115 |  |  | rs1050171 |  |  |  | rs1873778 |  |
| 24 | rs41115 |  |  | rs1050171 |  | rs3822214 |  | rs1873778 | rs1042522 |
| 25 | rs41115 |  |  | rs1050171 |  |  |  | rs1873778 | rs1042522 |
| 26 | rs41115 |  |  |  |  |  |  | rs1873778 | rs1042522 |
| 27 | rs41115 |  |  | rs1050171 |  |  |  | rs1873778 | rs1042522 |
| 28 | rs41115 |  |  | rs1050171 |  | rs3822214 |  | rs1873778 | rs1042522 |
| 29 | rs41115 |  |  | rs1050171 | rs1870377 | rs3822214 |  | rs1873778 | rs1042522 |
| 30 | rs41115 |  |  | rs1050171 | rs1870377 |  |  | rs1873778 | rs1042522 |
| 31 | rs41115 |  |  | rs1050171 |  |  |  | rs1873778 |  |
| 32 | rs41115 |  |  | rs1050171 |  |  |  | rs1873778 |  |
| 33 | rs41115 | rs1800056 |  | rs1050171 | rs1870377 |  |  | rs1873778 |  |
| 34 | rs41115 |  |  | rs1050171 |  | rs3822214 |  | rs1873778 |  |
| 37 | rs41115 |  |  | rs1050171 | rs1870377 |  |  | rs1873778 | rs1042522 |
| 36 | rs41115 |  |  | rs1050171 |  |  |  | rs1873778 | rs1042522 |
| 38 | rs41115 |  |  | rs1050171 |  | rs3822214 |  | rs1873778 |  |
| 39 | rs41115 |  |  | rs1050171 |  |  |  | rs1873778 |  |
| 40 | rs41115 |  |  | rs1050171 |  |  |  | rs1873778 |  |
| 41 | rs41115 |  |  |  |  |  |  | rs1873778 | rs1042522 |
| 42 | rs41115 |  |  | rs1050171 | rs1870377 |  |  | rs1873778 | rs1042522 |
| 43 | rs41115 |  |  | rs1050171 | rs1870377 |  |  | rs1873778 | rs1042522 |
| 44 | rs41115 |  |  | rs1050171 |  |  |  | rs1873778 | rs1042522 |
| 45 |  |  |  |  | rs1870377 |  | rs33917957 | rs1873778 | rs1042522 |
| 46 | rs41115 |  |  | rs1050171 | rs1870377 |  |  | rs1873778 | rs1042522 |
| 47 | rs41115 |  |  | rs1050171 | rs1870377 | rs3822214 |  | rs1873778 | rs1042522 |
| 48 | rs41115 |  |  | rs1050171 | rs1870377 |  |  | rs1873778 |  |
| 49 | rs41115 |  |  | rs1050171 |  |  |  | rs1873778 |  |
| 50 | rs41115 |  |  | rs1050171 | rs1870377 |  |  | rs1873778 |  |
| 51 | rs41115 |  |  | rs1050171 |  |  |  | rs1873778 |  |
| 52 | rs41115 |  |  | rs1050171 |  |  |  | rs1873778 |  |
| 53 | rs41115 |  |  |  | rs1870377 |  |  | rs1873778 |  |
| 54 | rs41115 |  |  | rs1050171 | rs1870377 |  |  | rs1873778 |  |
| 55 | rs41115 |  |  | rs1050171 |  |  |  | rs1873778 | rs1042522 |
| 56 | rs41115 |  |  | rs1050171 | rs1870377 |  |  | rs1873778 | rs1042522 |
| 57 | rs41115 |  |  | rs1050171 |  |  |  | rs1873778 | rs1042522 |
| 58 | rs41115 |  |  | rs1050171 |  |  |  | rs1873778 | rs1042522 |
| 59 | rs41115 |  |  | rs1050171 | rs1870377 |  |  | rs1873778 | rs1042522 |
| 60 | rs41115 | rs1800056 |  |  | rs1870377 |  |  | rs1873778 | rs1042522 |
| 61 | rs41115 |  |  | rs1050171 | rs1870377 |  |  | rs1873778 | rs1042522 |
| 62 | rs41115 |  |  | rs1050171 | rs1870377 | rs3822214 | rs33917957 | rs1873778 | rs1042522 |
| 63 | rs41115 |  |  | rs1050171 | rs1870377 |  |  | rs1873778 | rs1042522 |
| 64 | rs41115 |  |  | rs1050171 | rs1870377 | rs3822214 |  | rs1873778 |  |
| 65 | rs41115 |  |  |  |  |  |  | rs1873778 | rs1042522 |
| 66 |  |  |  | rs1050171 |  |  |  | rs1873778 | rs1042522 |
| 67 |  |  | rs1801023 | rs1050171 |  |  |  | rs1873778 |  |
| 68 | rs41115 |  |  | rs1050171 |  |  |  |  | rs1042522 |
| 69 |  |  |  | rs1050171 | rs1870377 |  |  | rs1873778 | rs1042522 |
