# Supplementary material for: Dissecting Genomic Aberrations in Myeloproliferative Neoplasms by Multiplex-PCR and Next Generation Sequencing
Source: PLoS One. 2015 Apr 20;10(4):e0123476. doi: 10.1371/journal.pone.0123476 (PMC4404337; doi:10.1371/journal.pone.0123476)
Supplement: S2 Table — List of regions which were analyzed by base space software (illumina) which showed amplicon regions with no amplification product. (DOCX) [file pone.0123476.s002.docx]

**Supplement 2 (Table)**:

| Primer with no amplificat: |
| --- |
| PTEN7.chr10.89717615.89717772_tile_1.PTEN4.chr10.89711893.89711900_tile_1.2 |
| PTEN7.chr10.89717615.89717772_tile_1.PTEN3.chr10.89685307.89685307_tile_1.2 |
| GNAQ_6.chr9.80343430.80343583_tile_1.GNAQ_7.chr9.80336240.80336429_tile_3.2 |
| GNA11_7.chr19.3120987.3121177_tile_1.GNA11_4.chr19.3114942.3115070_tile_2.2 |
| GNA11_6.chr19.3119204.3119357_tile_2.GNA11_7.chr19.3120987.3121177_tile_2.2 |
