# Supplementary material for: Dissecting Genomic Aberrations in Myeloproliferative Neoplasms by Multiplex-PCR and Next Generation Sequencing
Source: PLoS One. 2015 Apr 20;10(4):e0123476. doi: 10.1371/journal.pone.0123476 (PMC4404337; doi:10.1371/journal.pone.0123476)
Supplement: S3 Table — List of primers with amplification of pseudogene regions. First column shows primer name (basespace, illumina), second column shows pseudogene location (chromosome) (DOCX) [file pone.0123476.s003.docx]

**Supplement 3 (Table)**:

| Amplicons in pseudogenes: | Location |
| --- | --- |
| GNAQ_5.chr9.80409379.80409508_tile_1.GNAQ_7.chr9.80336240.80336429_tile_1.2 | chr 2 |
| GNAQ_6.chr9.80343430.80343583_tile_1.GNAQ_7.chr9.80336240.80336429_tile_3.2 | chr 2 |
| GNAQ_7.chr9.80336240.80336429_tile_1.2 | chr 2 |
| GNAQ_7.chr9.80336240.80336429_tile_2.GNAQ_5.chr9.80409379.80409508_tile_2.2 | chr 2 |
| GNA11_6.chr19.3119204.3119357_tile_2.GNA11_7.chr19.3120987.3121177_tile_2.2 | chr 7 |
| GNA11_7.chr19.3120987.3121177_tile_1.GNA11_4.chr19.3114942.3115070_tile_2.2 | chr 7 |
| IDH1_1_2.chr2.209113112.209113113_tile_1.2 | chr 6 |
| PIK3CA12.chr3.178938860.178938860_tile_1.2 | chr 22 |
| PIK3CA4_11.chr3.178936074.178936095_tile_1.2 | chr 22 |
| PTEN13.chr10.89720716.89720852_tile_1.2 | chr 9 |
