# Supplementary material for: Dissecting Genomic Aberrations in Myeloproliferative Neoplasms by Multiplex-PCR and Next Generation Sequencing
Source: PLoS One. 2015 Apr 20;10(4):e0123476. doi: 10.1371/journal.pone.0123476 (PMC4404337; doi:10.1371/journal.pone.0123476)
Supplement: S4 Table — (DOCX) [file pone.0123476.s004.docx]

**Supplement 4 (Table):**

| ABL1 | EGFR | GNAS | MLH1 | RET |
| --- | --- | --- | --- | --- |
| AKT1 | ERBB2 | HNF1A | MPL | SMAD4 |
| ALK | ERBB4 | HRAS | NOTCH1 | SMARCB1 |
| APC | FBXW7 | IDH1 | NPM1 | SMO |
| ATM | FGFR1 | JAK2 | NRAS | SRC |
| BRAF | FGFR2 | JAK3 | PDGFRA | STK11 |
| CDH1 | FGFR3 | KDR | PIK3CA | TP53 |
| CDKN2A | FLT3 | KIT | PTEN | VHL |
| CSF1R | GNA11 | KRAS | PTPN11 |  |
| CTNNB1 | GNAQ | MET | RB1 |  |
