# Supplementary material for: Dissecting Genomic Aberrations in Myeloproliferative Neoplasms by Multiplex-PCR and Next Generation Sequencing
Source: PLoS One. 2015 Apr 20;10(4):e0123476. doi: 10.1371/journal.pone.0123476 (PMC4404337; doi:10.1371/journal.pone.0123476)
Supplement: S5 Table — List of primers used for validation of detected variants by conventional sanger sequencing (upper part) and allele specific PCR (lower part, according to Jones et al., BLOOD, 2005) (DOCX) [file pone.0123476.s005.docx]

**Supplement 5 (Table):**

| **Primer pairs for conventional Sanger Sequencing:** |  |  |  |
| --- | --- | --- | --- |
| CSF1R_Forward | TCCCACCCTCAGGACTATACCAATCT | CSF1R_Rev | CCCTGTCGTCAACTCCTCAGCAGAACT |
| ERBB2_ Forward | TCTGCTCCTTGGTCCTTCACCTAACCT | ERBB2_Rev | CCAGGGGATGAGCTACCTGGAGGATGT |
| IDH1_ Forward | GAGGGTTGAGGAGTTCAAGTTGAAACA | IDH1_Rev | ATGACTTACTTGATCCCCATAAGCATG |
| KIT_ Forward | TCCTGCCAAAGTTTGTGATTCCAC | KIT_Rev | GTGACATGGAAAGCCCCTGTTTCATA |
| KRAS_ Forward | TCTTTCCCAGAGAACAAATTAAAAGAGT | KRAS_Rev | TTTCAGTGTTACTTACCTGT |
| MET_ Forward | TGCAAAACCAAAAATAAACAACAATGTC | MET_Rev | TAGGCTTGTAAGTGCCCGAAGTGTAA |
| NRAS_ Forward | GGGTTTTCATTTCCATTGATTATAGAAAG | NRAS_Rev | TGGTTCTGGATTAGCTGGATTGTCAG |
|  |  |  |  |
| **Primer for allele specific PCR (JAK2 validation):** |  |  |  |
| forward outer | TCCTCAGAACGTTGATGGCAG |  |  |
| reverse outer | ATTGCTTTCCTTTTTCACAAGAT |  |  |
| forward wild-type-specific | GCATTTGGTTTTAAATTATGGAGTATaTG |  |  |
| reverse-mutant-specific | GTTTTACTTACTCTCGTCTCCACAaAA |  |  |
