# Supplementary figures and images for: Dissecting Genomic Aberrations in Myeloproliferative Neoplasms by Multiplex-PCR and Next Generation Sequencing
Source: PLoS One. 2015 Apr 20;10(4):e0123476. doi: 10.1371/journal.pone.0123476 (PMC4404337; doi:10.1371/journal.pone.0123476)

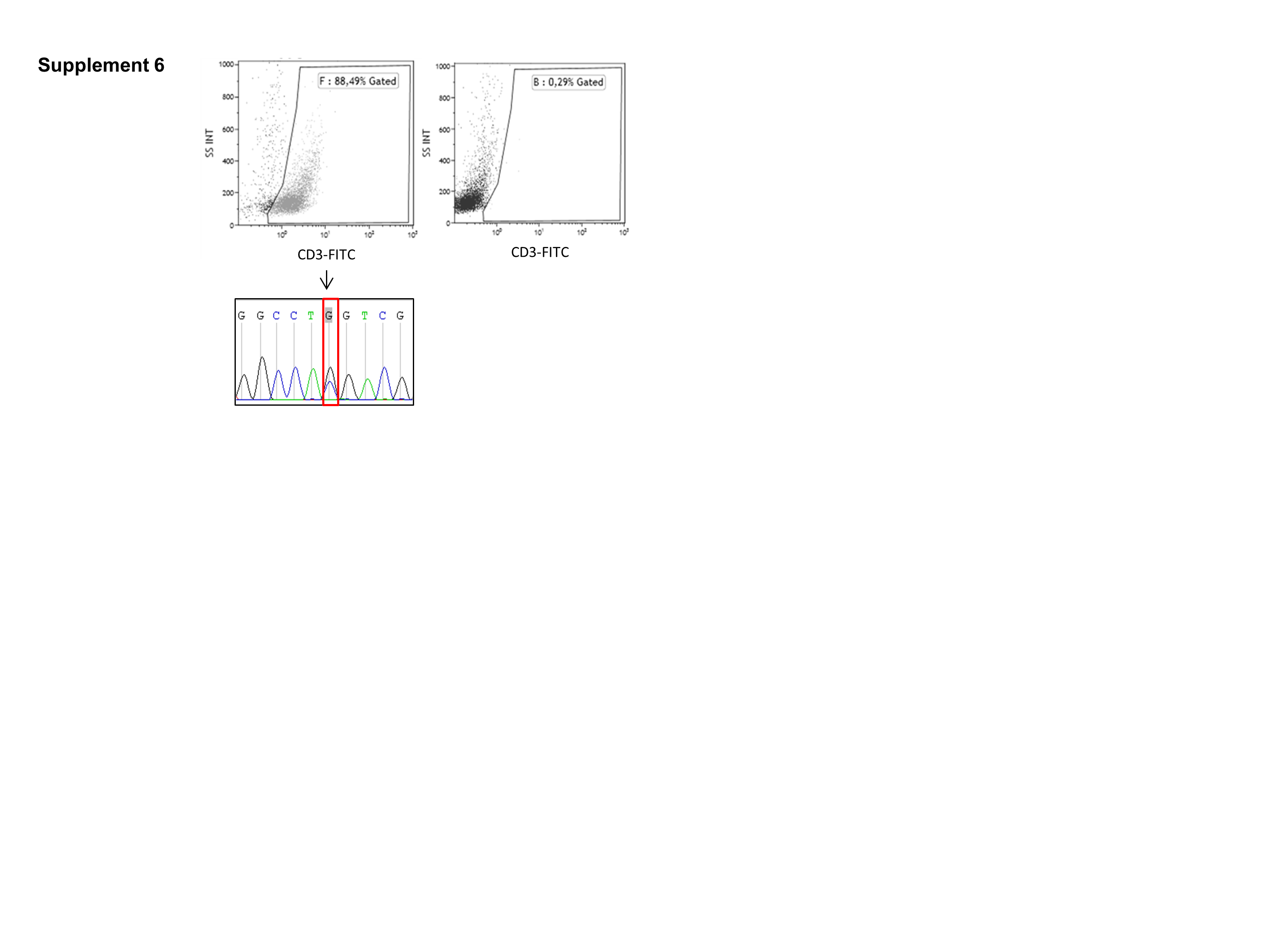

Supplement: S1 Fig — The previously described c-kit V530I SNP was present in total PB-derived as well as CD3+ selected T cells from a patient with CML. CD3 MACS-sorted cells (left) and CD3-depleted Cells (right) were stained as control using CD3-FITC antibody. Sanger sequencing was performed using CD3 pos T-cell population (lower panel) (TIF) [file pone.0123476.s006.tif]

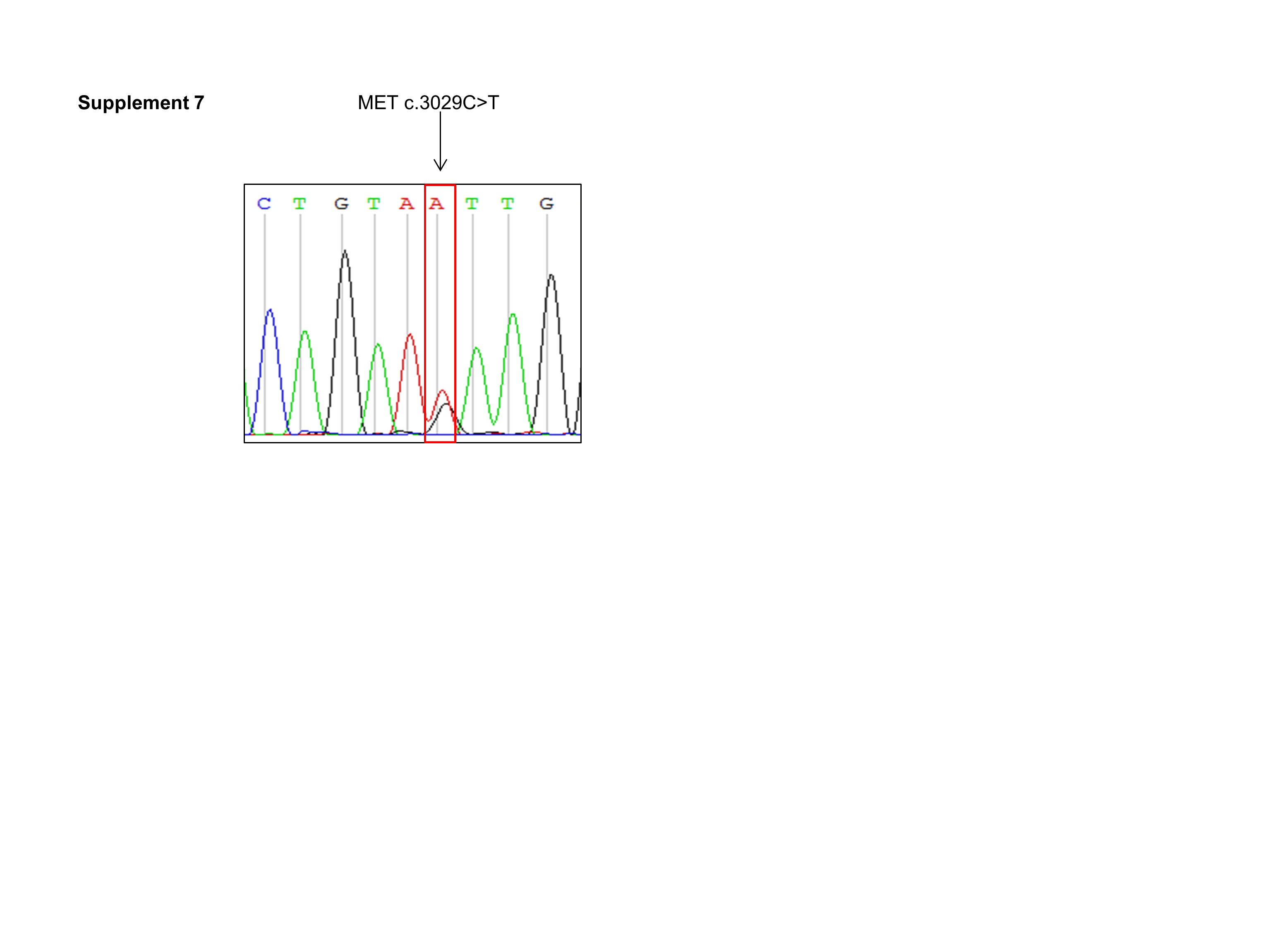

Supplement: S2 Fig — (TIF) [file pone.0123476.s007.tif]

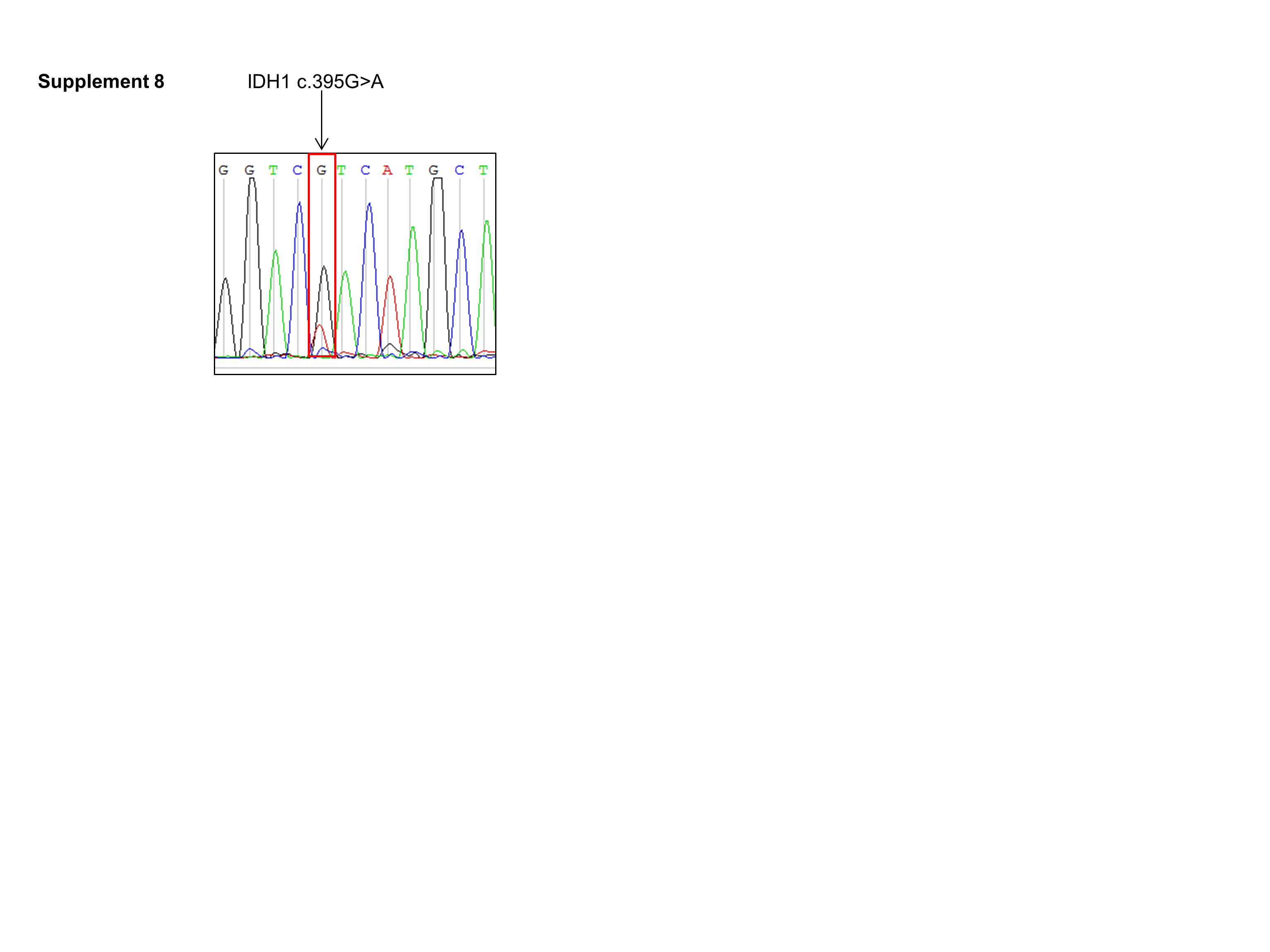

Supplement: S3 Fig — (TIF) [file pone.0123476.s008.tif]

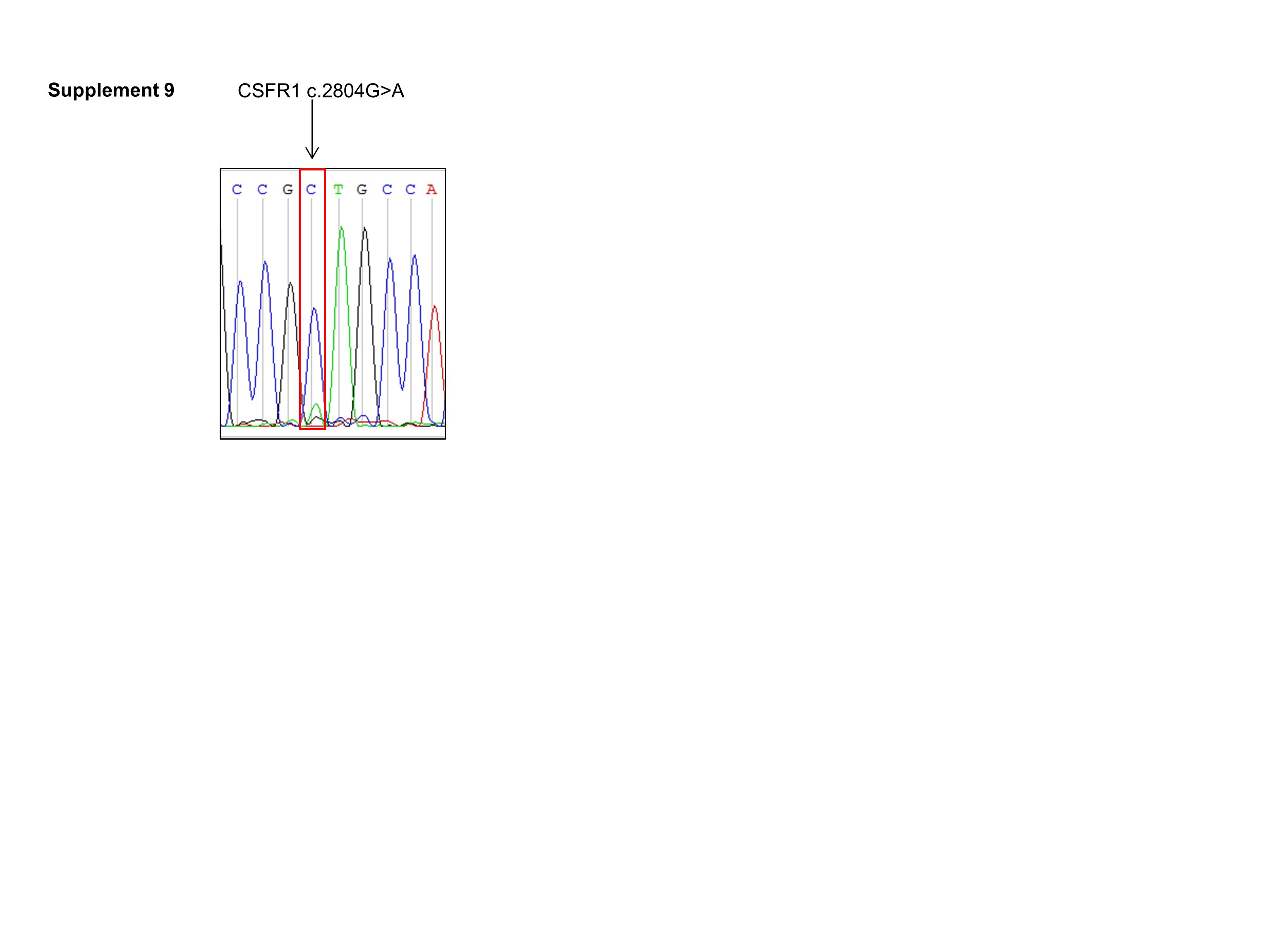

Supplement: S4 Fig — (TIF) [file pone.0123476.s009.tif]
