# Supplementary material for: Dissecting Genomic Aberrations in Myeloproliferative Neoplasms by Multiplex-PCR and Next Generation Sequencing
Source: PLoS One. 2015 Apr 20;10(4):e0123476. doi: 10.1371/journal.pone.0123476 (PMC4404337; doi:10.1371/journal.pone.0123476)
Supplement: S1 File — Additional methods not mentioned in the methods part of the manuscript. (DOCX) [file pone.0123476.s010.docx]

**Supplement methods:**

**Validation of germline SNV:**

The KIT c.1588G>A (V530I) variant was analyzed for their respective germline variant status. For this, peripheral blood mononuclear cells (MNCs) were isolated using density gradient centrifugation (Ficoll, GE Healthcare) and MNCs were stained and purified using anti-CD3 MicroBeads (Miltenyi Biotec). Purity control was performed by FACS. Sequence analysis was conducted by Sanger sequencing (Supplement 6).

**Validation by conventional Sanger sequencing**

Several detected variants (CSF1R, JAK2, IDH1, KIT, KRAS, MET) were validated by the use of Sanger sequencing after PCR amplification (examples see Supplement 7-9). Used primers are listed in Supplement 5.
